# Restorative treatment in a case of amelogenesis imperfecta and 9-year follow-up: a case report

**DOI:** 10.1186/s13005-020-00243-1

**Published:** 2020-11-19

**Authors:** Martin M. I. Sabandal, Till Dammaschke, Edgar Schäfer

**Affiliations:** 1 Central Interdisciplinary Ambulance in the School of Dentistry, University of Münster, Albert-Schweitzer-Campus 1, Gebäude W30, Waldeyerstr. 30, 48149 Münster, Germany; 2 Department of Periodontology and Operative Dentistry, University of Münster, Münster, Germany

**Keywords:** *Amelogenesis imperfecta*, Direct restoration, Follow-up, Restorative treatment

## Abstract

**Background:**

*Amelogenesis imperfecta* is a hereditary malformation showing various manifestations regarding enamel dysplasia. This case report shows a 9-year follow-up after restorative treatment of a 16-year old female patient affected by a hypoplastic type of *amelogenesis imperfecta*. The caries-free, hypersensitive teeth of the patient were restored by direct dentin adhesive composite restorations performed in total etch technique.

**Case presentation:**

After rehabilitation the patient reported a marked improvement of the mastication ability and quality of life especially during food intake. Accumulation of plaque was reduced and the ability to perform adequate oral hygiene was improved. During follow-up of 9 years recurring secondary caries and debonding of fillings were recognized and retreated.

**Conclusions:**

The retrospective assessment exhibits that the performed restorative treatment prolonged the time until further treatment has to be considered, such as prosthetic treatment.

## Background

*Amelogenesis imperfecta* (AI) is a genetic derived development disorder of the ameloblasts during the formation of enamel. The disease is characterized by visible malformation of the enamel in a varying degree of the whole permanent and deciduous dentition, respectively [1, 2].

The prevalence of AI ranges from 1:700 to 1:14,000 [1]. Because of the various manifestations of AI Weinmann et al. suggested in 1945 a classification [3] that has been modified and adapted during the following years depending on the diagnostic possibilities. A useful classification was proposed by Witkop [2], which is based on a division of the manifestation of AI into 4 main groups with further subdivisions [2].

Type I -hypoplastic.

IA -hypoplastic, pitted autosomal dominant.

IB -hypoplastic, local autosomal dominant.

IC -hypoplastic, local autosomal recessive.

ID -hypoplastic, smooth autosomal dominant.

IE -hypoplastic, smooth X-linked dominant.

IF -hypoplastic, rough autosomal dominant.

IG -enamel agenesis, autosomal recessive.

Type II -hypomaturation.

IIA -hypomaturation, pigmented autosomal recessive.

IIB -hypomaturation, X-linked recessive.

IIC snowcapped teeth, X-linked.

IID autosomal dominant?

Type III -hypocalcified.

IIIA -autosomal dominant.

IIIB -autosomal recessive.

Type IV -hypomaturation-hypoplastic with taurodontism.

IVA -hypomaturation-hypoplastic with taurodontism, autosomal dominant.

IVB -hypoplastic-hypomaturation with taurodontism, autosomal dominant.

**Fig. 1:**
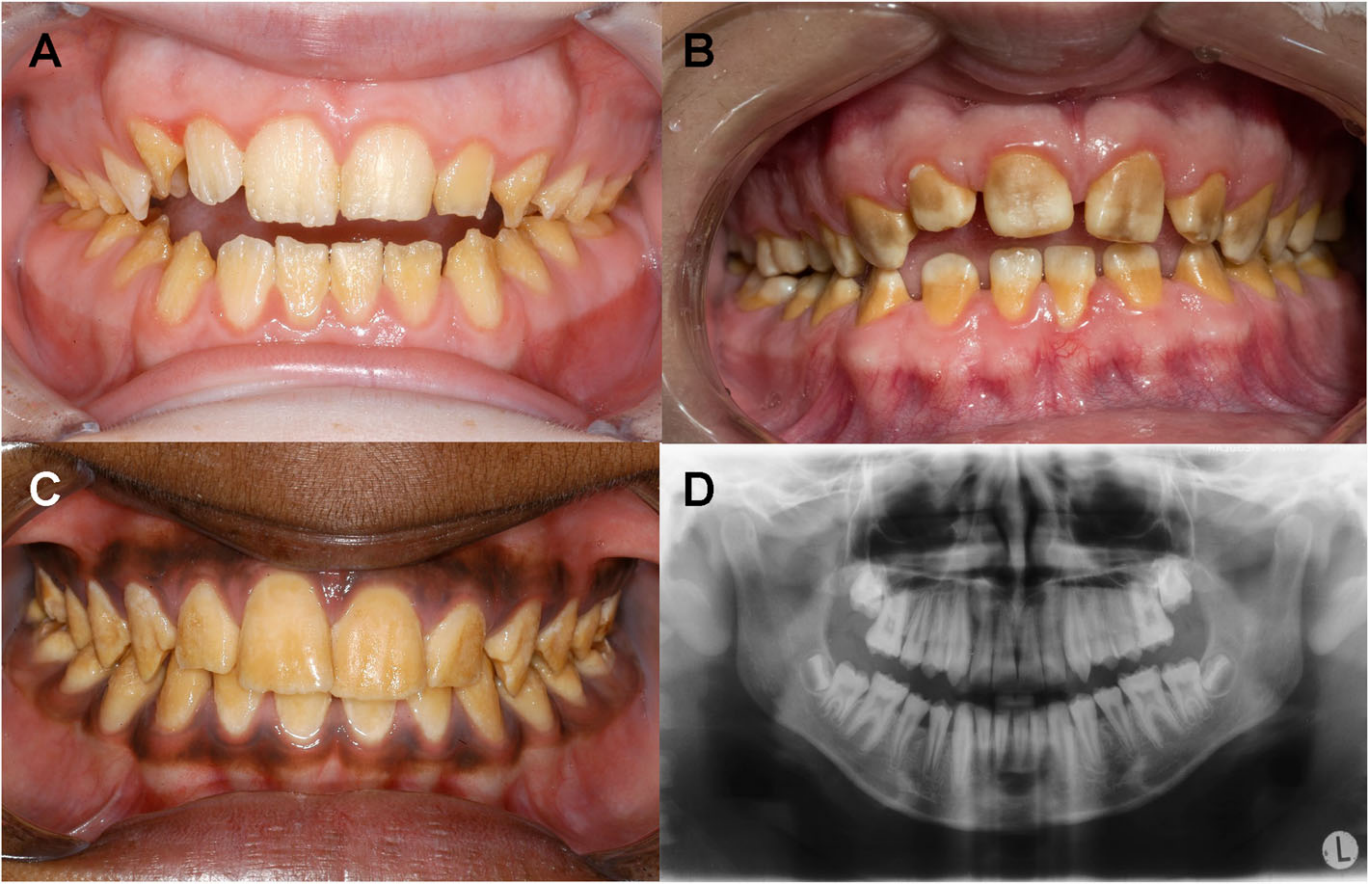
Types of amelogenesis imperfecta in the anterior aspect; hypoplastic type of AI = **a**, hypomaturated type of AI=**b**, hypocalcified type of AI=**c**; orthopantomogram shows hypomaturated-hypoplastic with taurodontism AI = **d**

Especially the hypoplastic type of AI shows an increased hypersensitivity upon chemical and physical stimuli from tooth eruption onwards. The subtypes of the hypoplastic AI are characterized by rough dental surfaces due to the irregularly lacking enamel areas [4, 5]. These findings were originated by the partial or complete missing of enamel coverage and often dentin areas are exposed [1, 2, 6–9] (Fig. 1a). In the hypoplastic type of AI enamel and dentin can be sophisticated by a regular radiographic contrast [2], with marked reduced enamel thickness [2, 10–12].

AI of hypomaturated type summarizes malformation of the enamel with reduced hardness, normal crown shape and enamel thickness [9]. The colour of the altered enamel shows a mottled pattern and discoloured appearance [13] (Fig. 1b). Often chipping of hypomaturated enamel is evident [2]. The radiopacity of both enamel and dentin is nearly equal [2]. In AI of hypomaturated type a X-linked recessive genetic trait has been described [2].

The third main group of AI shows an autosomal trait of hypocalcified AI alterations of enamel [2]. The dysplastic enamel is of regular shape and normal thickness regarding the enamel coverage [9]. The visible enamel shows a discoloured orange-yellow surface pattern [9] (Fig. 1c). The malformed enamel consists of a poorly calcified matrix resulting in a higher radiolucency of the enamel compared to the underlying dentin [2].

The fourth main group of enamel malformation is type IV of combined hypomaturated and hypoplastic enamel associated with taurodontism [2]. Predominantly the altered enamel shows hypoplastic patterns with partly hypomaturated areas. The radiolucency of the enamel is slightly decreased or nearly equal compared to dentin [2] (Fig. 1d).

Of all types of AI the hypoplastic type is with 61.2% the most frequent type, the hypomaturated type follows with 32.2% and types III and IV combined show a rate of 3.2% of all cases affected by AI [14]. Due to several forms of AI showing autosomal recessive traits the original classification of Witkop [2] was revisited by Nusier et al. [15].

Beside malformation of the enamel also skeletal alterations especially of the jaws may occur. Approximately 24% of all investigated subjects affected by AI show an anterior open bite (AOB) [16, 17]. Additionally, subjects with AOB display a marked discrepancy of the vertical dimension. About 20% of subjects affected by AI without AOB also show an alteration of the vertical dimension [17]. The multiple findings seen in subjects affected by AI require an interdisciplinary treatment by orthodontic, prosthetic, restorative and in some cases surgical treatment [7].

All alterations and types of AI are possible in combination with different genetic traits.

## Case report

### Diagnosis and aetiology

**Fig. 2:**
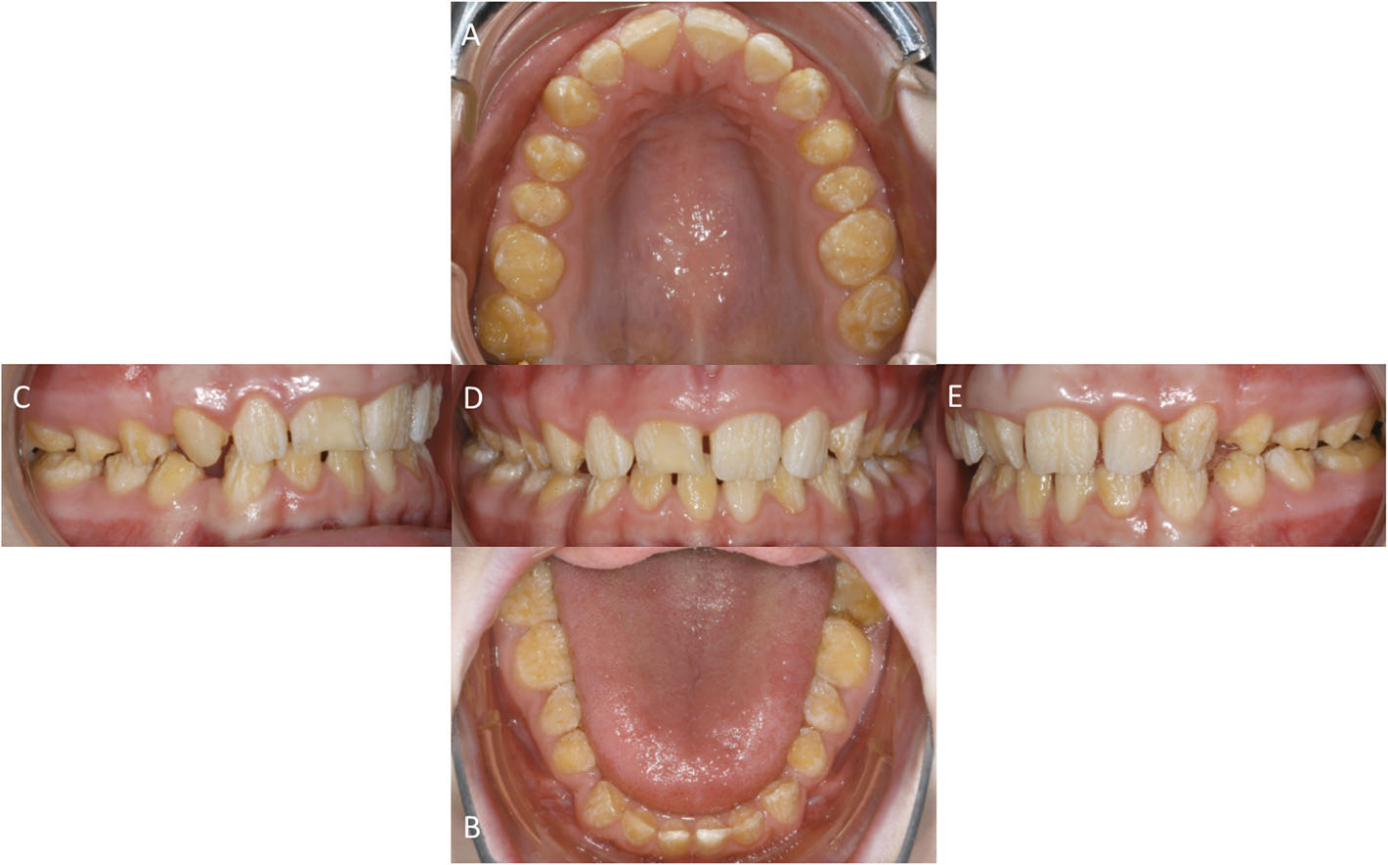
Occlusal view of the upper jaw = **a** and lower jaw = **b**. Enface view anterior = **d**, right = **c** and left = **e** all prior to treatment

In april 2010 a 16-year old female patient showed herself in the Central Interdisciplinary Ambulance in the School of Dentistry, University of Münster for consultation. Her dentist intended to perform a prosthetic full crowning of all teeth due to the generally hypoplastic enamel (Fig. 2). The parents of the patient asked for the necessity of prosthetic treatment at that point of time. An orthodontic treatment to regulate a minor existing malocclusion was completed at that time. Her dentist recognized the alteration of the teeth upon eruption of the deciduous teeth and later the enamel malformation was also evident in the permanent teeth.

The medical history was unremarkable. Since the diagnosis of AI the patient got frequent appointments at her dentist for high fluoride varnish and professional dental care every 6 weeks. Beside that the patient performed an in-house fluoridation once a week with high dose fluoride and daily use of fluoride containing tooth paste for tooth brushing.

A familial clustering was reported by the patient and her parents. Affected subjects were known in different branches of the family. Her sister was also affected in an equal manner. No further dental findings or caries were recognizable.

The suspected diagnosis was *amelogenesis imperfecta* type I according to the classification of Witkop [2]. The diagnosis was based on the phenotype and family history of the relevant findings of amelogenesis imperfecta.

Due to the partially exposed dentin and lack of regularly formed overlying enamel the teeth of the patient showed hypersensitivity upon chemical and physical stimuli. Especially sweet food and beverages caused increased disturbance by pain. As a consequence, the patient altered her alimentary habits and avoided the consumption of all kind of food that caused pain.

Beside the limitation of food consumption, the major discomfort was the reduced esthetic appearance due to the malformed tooth shape and appearance of the visible anterior teeth of the upper and lower jaw. Due to the malformed tooth shape and the discolouration the patient reported negative social influence when trying to hide the altered teeth during talking and laughing to each other.

Due to the well done frequently appointments and fluoridation at her dentist and the good oral hygiene no signs of gingivitis were present. The only filling was found within the lower left second molar.

### Findings

On dental examination a complete adult dentition without the upper and lower third molars was recognizable. Due to a former defect the last lower left second molar was restored with a composite resin filling. No other restorations were recognizable.

The shape of the visible clinical crowns of the permanent dentition was altered by the hypoplastic enamel. As reported before the patient mentioned similar alteration within the deciduous dentition.

### Radiographic findings

**Fig. 3:**
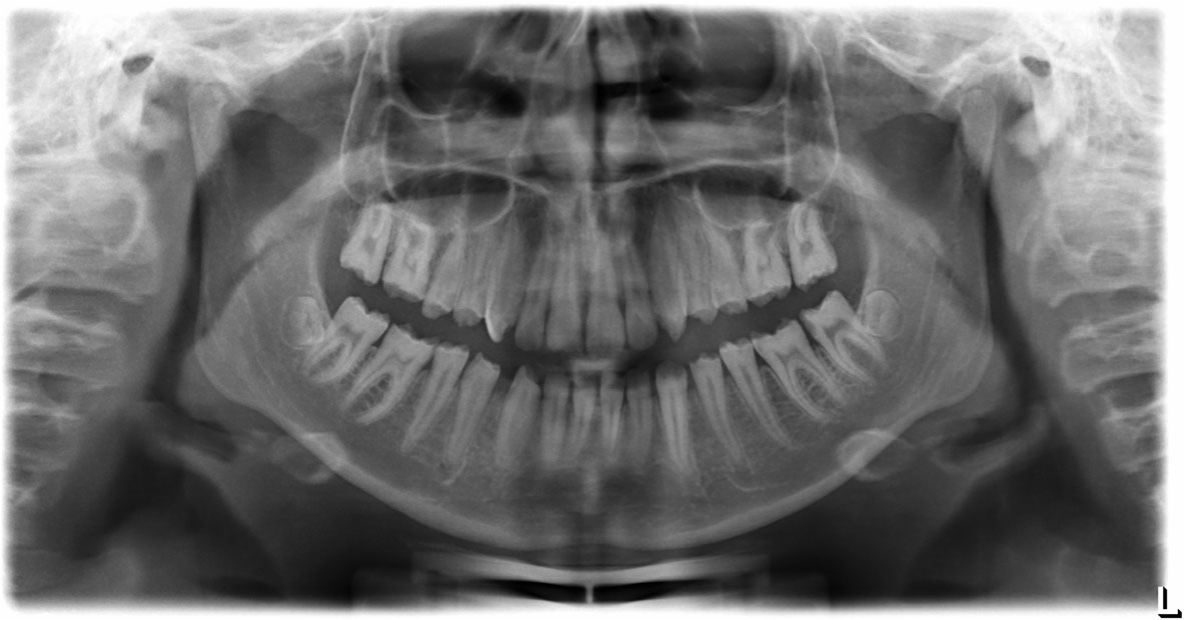
Orthopantomogram prior to treatment

Prior to the suggested treatment an orthopantomogram was done by the former dentist (Fig. 3). A complete permanent dentition was recognizable, but the lower third molars were still unerupted. The orthopantomogram revealed that there was an agenesis of the upper left and right third molars. The residual covering enamel showed no reduced radiolucency but reduced thickness compared to the underlying dentin. No radiolucency corresponding to carious lesions was found. The pulp chambers especially of the upper and lower first and second molars showed reduced volumes compared to the mean volume of such teeth in patients of the same age not affected by AI. The length of the tooth roots appeared to be in normal range, while the interdental spaces, especially in the lower jaw appeared enlarged.

### Prior performed orthodontic treatment

In advance of the first presentation in the central interdisciplinary ambulance an orthodontic treatment was performed by the orthodontist near the patient’s home. Due to the hypoplastic and partly aplastic enamel the orthodontic treatment was done by using the Invisalign system (Align Technology Switzerland GmbH, Rotkreuz, Switzerland). The aim of the orthodontic treatment was a pre-prosthetic alignment of the teeth suggested by the former dentist. The upper and lower dental arches were harmoniously shaped, and a slight gap position of the approximal spaces was also adjusted. However the designated prosthetic restoration was not carried out.

### Restorative treatment

Cast models were made after dental impressions with alginate to preserve the initial oral situation prior to further treatment. To determine possible alterations of the esthetic aspects a measurement of the upper face third from hairline to glabella (5 cm), middle face third from glabella to subnasal (5 cm) and lower face third in maximum intercuspidation from subnasal to menton (4.7 cm) was done. Additionally, a deep bite of 5 mm was measured, whereas an overbite of 1–2 mm can usually be measured; the difference corresponds approximately to the sum of the missing enamel thickness, which in non-pathological cases is found in the posterior region [18]. The vertical dimension was not elevated in agreement with the patient.

The restoration of all teeth of the upper and lower jaw except upper left lateral incisor and the lower left premolars was done by direct composite resin fillings. Due to existing occlusal contacts located in the areas of residual enamel within the maximum intercuspidation the occlusal relation was secured without further alterations and without the need of elevating the vertical dimension. Five appointments were scheduled for the entire treatment. During each appointment the teeth of a quadrant and the anterior upper incisors were treated successively. During each appointment the restoration of the teeth was done after adaption of rubber dam and additionally insertion of a braided retraction thread of size 00 (Ultrapak Cord, Ultradent, South Jordan, UT, USA) impregnated in Orbat (lege artis Pharma, Dettenhausen, Germany) in the sulcus gingivalis in order the prevent efflux of sulcus fluid and blood during restorative therapy.

After total etch of the tooth surfaces by phosphoric acid (38%) (Orbis Dental, Münster, Germany) the chosen dentin adhesive Optibond FL (Kerr, Orange, CA, USA) was applied to the etched surfaces according to the manufacturer’s specifications to ensure an appropriate bonding of the composite resin restoration to the residual enamel and underlying dentin. For restoration of the hypoplastic teeth the nanofilled hybrid composite resin Grandio (VOCO, Cuxhaven, Germany) was used. In agreement with the patient the molars and canines were restored with the shade Vita A3 whereas the premolars and incisors with the shade Vita A2.

The exposed dentin and residual enamel of the dysplastic teeth was covered in anatomic shape by the use of composite resin restoration and light cured with a LED polymerization light at wavelength of 450 to 490 nm (SmartLite PS, Dentsply, Konstanz, Germany) for 20 s per increment. The restoration of the occlusal, buccal and oral surfaces was done by free-hand modelling whereas the proximal parts of the teeth were modelled with matrices (Orbis Dental, Münster, Germany) and interdental wedges (Kerr Hawe, Bioggio, Switzerland).

After completion of the restorations all restored surfaces were contoured, the occlusion was corrected by yellow coded diamond burs (Brasseler, Lemgo, Germany) and polished with Identoflex Composite Polishers and Occlubrush (both KerrHawe). The treatment of the upper jaw required 3 appointments whereas the treatment of the lower jaw required 2 appointments.

**Fig. 4:**
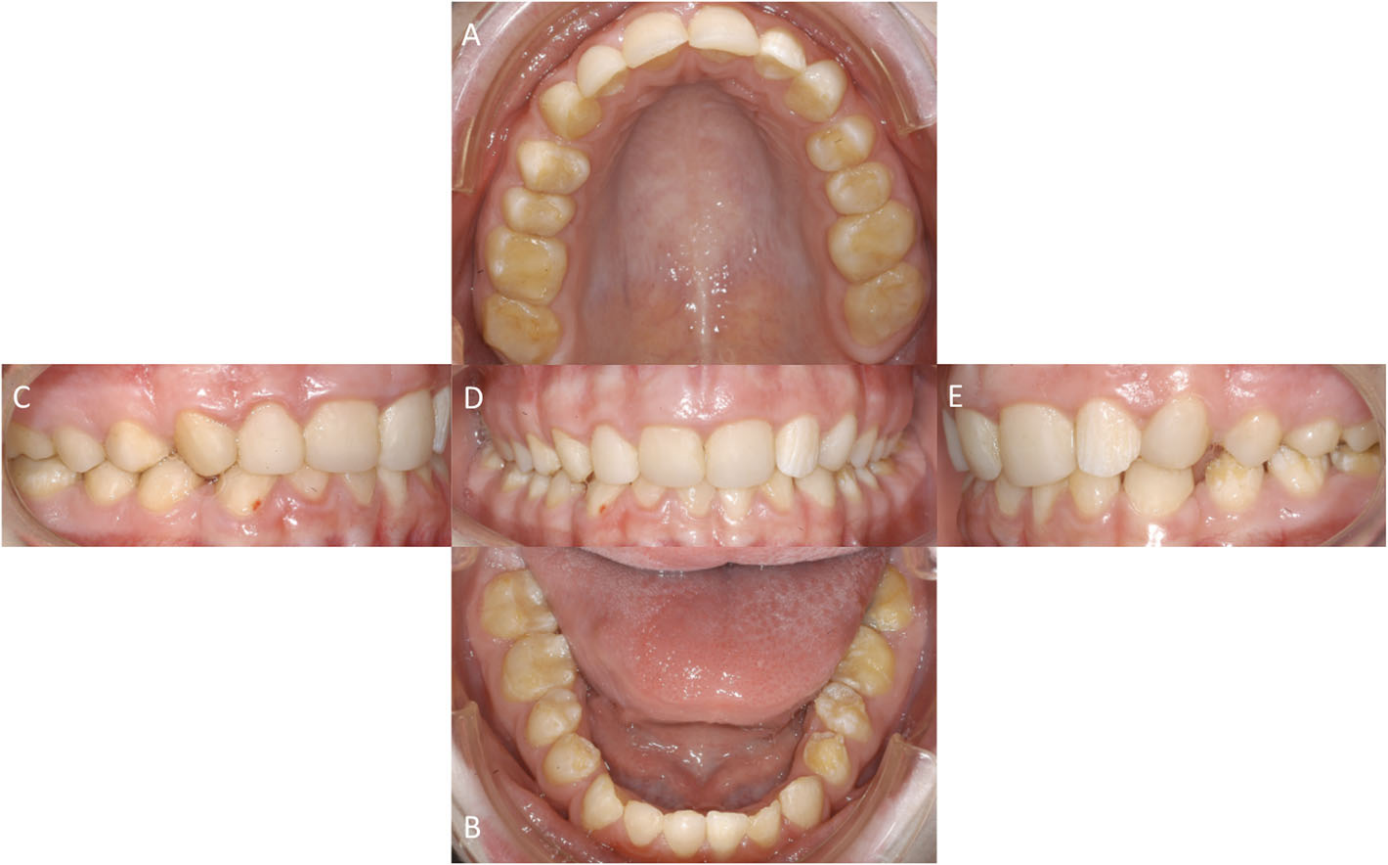
Occlusal view of the upper jaw = **a** and lower jaw = **b**. Enface view anterior = **d**, right = **c** and left = **e** all after restorative treatment

In agreement with the patient, some teeth (upper left lateral incisor and the lower left premolars) and some lingual and palatal surfaces were not treated due to an existing enamel layer. Due to insufficient distal margin the filling of the second left lower molar was retreated (Fig. 4).

After the completion of the treatment the patient got intensive oral hygiene instructions. The fluoridation schema containing fluoride varnish in the dental office once every 6 weeks, application of high dose fluoride formulation once a week with Elmex Gelée (GABA, Lörrach, Germany) or Sensodyne PROSCHMELZ Fluorid Gelée (GlaxoSmithKline, Bühl, Germany) and daily oral hygiene with fluoride containing tooth paste, toothbrush and dental floss was recommended. Beside also appointments for dental control every 3 months in the dental office were recommended.

After carrying out the restoration the patient decided to undertake the recommended regular appointments and dental care at her family dentist near to her home in order to avoid frequently long travels for appointments to the School of Dentistry of Münster.

### Follow-up

Seven months after completion of the restoration the first follow-up was performed. All restorations were sufficient and no discolouration was visible. Solely a slight bleeding on probing located in the sulcus gingivalis of the right lower canine was visible but no other signs of gingival inflammation were recognizable.

After about 2 years (28 months) without regular appointments for control at the School of Dentistry in Münster the patient showed again for retreatment. Some restorations showed secondary caries especially located in the interdental spaces of the both upper first molars and the second left lower premolar. The defects of the teeth were again treated in the same manner as described with dentin adhesive fillings. The composite resin used was Grandio SO (VOCO, Cuxhaven, Germany).

**Fig. 5:**
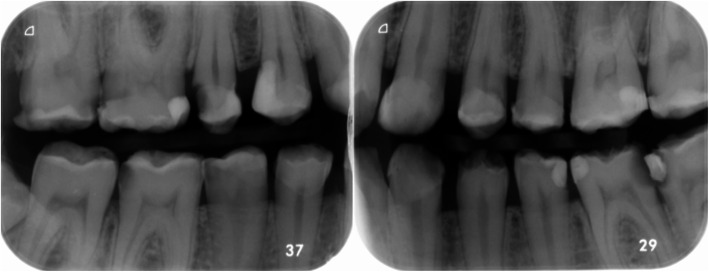
Bitewings right and left taken at 46 months followup

About 4 years (46 months) after initial treatment the patient showed herself for dental control. Bitewing radiographs of the right and left side were taken (Fig. 5). Some restorations showed radiolucency of the margins due to secondary caries and caries without association to former restorations especially within the interdental spaces. A restorative therapy of the affected teeth was done in the same manner as initially performed with total etch technique, dentin adhesive and composite resin (Tetric Evoceram, Ivoclar Vivadent, Schaan, Lichtenstein). Treatment was necessary to the upper right jaw (second premolar to the second molar), the upper left jaw (first premolar and second molar), the lower left jaw (second premolar to the second molar) and the lower right jaw (second premolar and fist molar).

Sixty-eight months (5.5 years) after restoration the patient showed herself for another follow-up control. Due to secondary caries the restoration of the second lower right molar showed an insufficient margin. A retreatment was done with total etch technique, dentin adhesive and composite resin (IPS Empress Direct, Ivoclar Vivadent).

**Fig. 6:**
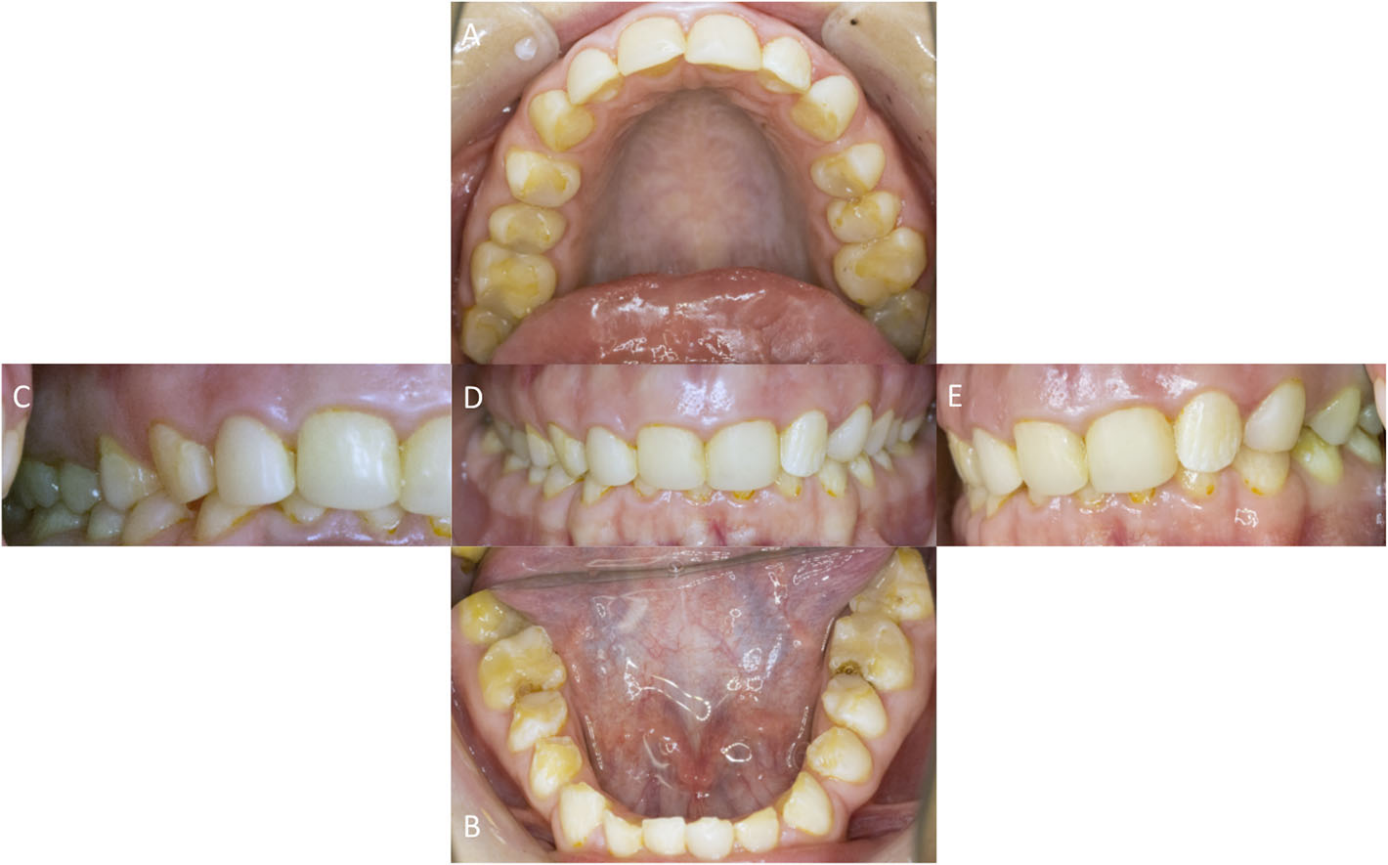
Occlusal view of the upper jaw = **a** and lower jaw = **b**. Enface view anterior = **d**, right = **c** and left = **e** all at 9-year follow-up

The last follow-up was done about 9 years (110 months) after initial treatment. During the appointment some composite resin fillings showed insufficient margins (second upper left premolar, first lower left molar, first and second lower right molar) and had to be retreated (Fig. 6).

## Discussion

The present findings show generally malformed teeth regarding tooth shape and tooth colour of both the permanent and deciduous dentition and increased sensitivity upon physical and chemical stimuli. These findings correspond to the main complaints reported from patients affected by AI [9, 11–13]. The altered tooth shape and smaller appearance of all teeth caused by the lack of enamel is accompanied by exposed dentin, which may explain increased sensitivity of the dysplastic teeth.

Likewise, 90% of all patients affected by AI [19], like in the present case, report a decreased quality of life due to discolouration and increased sensitivity. This is in agreement with the findings of Parekh et al. 2014 [19] in as far as 77% of the patients affected by AI mentioned as a main compliant the discolouration of the teeth [19] and 74% suffered from the increased sensitivity [19].

Different treatment concepts considering the specific findings and characteristics of AI have been reported [20]. Subjects affected by AI require a multidisciplinary dental treatment approach when various dental and skeletal findings are evident and a proper treatment and restoration is desired [20]. In 24% [14] to 64% [7] of subjects affected by AI an anterior open bite and further malocclusions [17] have been reported.

Some studies investigated possible skeletal and orthodontic alterations in subjects affected by AI [7, 17], however in the present case no anterior open bite or other malocclusions were evident. But an anterior deep bite due to an increased overbite of 5 mm distance between the incisal edges of the upper and lower central incisors was recognizable. In comparison to the frequently reported multidisciplinary dental treatments consisting of orthodontic and/or surgical treatment prior to prosthetic restoration in subjects affected by AI [7, 21], no necessity of such treatments was evident in the present case. Although no major skeletal changes could be detected, the vertical dimension was reduced as expected due to the lack of enamel [18]. Compared to the upper and middle face third the lower face third was reduced by approximate 3 mm which corresponds to the average thickness of the missing enamel layer [18]. The reconstruction was carried out after minor orthodontic treatment combined with a slight enlargement of the approximal spaces.

Direct composite resin restorations were chosen to improve the quality of life by reducing the patient main compliant (reduced esthetic appearance, increased sensitivity upon physical and chemical stimuli) without an increased risk of pulp exposure and irreversible irritation of the pulp tissue during preparation and treatment. Especially the extension of the pulp chamber with prominent pulp horns in younger patients should be taken into account [22] in case of extensive restorations. Extensive full-crown restorations of the whole dentition should be avoided in young patient whenever possible.

In the present case a minimal invasive treatment of the patient was intended to improve the comfort of mastication, to decrease the hypersensitivity upon thermal and chemical stimuli and to prolong time until possible further treatments has to be considered. As recent studies reported [8, 21] the hypersensitivity upon thermal and chemical stimuli was decreased immediately after restoration. As a side effect of the minimal invasive restorative treatment the formerly rough hypoplastic dental surfaces were smoothened by the application of composite resin restoration. Additionally, the plaque accumulation was decreased compared to the pre-existing status and an improvement of the ability of oral hygiene was reported.

At the patient’s request, the follow-up appointments were scheduled with the family dentist, who did not perform any further restorative treatments during the follow-ups. About every 2 years the patient made an appointment in the School of Dentistry of Münster for check-up. When showing for an appointment frequently reconstructive treatments due to secondary caries were necessary. The necessity of recurrent retreatment of dentin adhesive fillings described in the present case agrees with the in general reduced longevity of dental restorations in subjects affected by AI [23]. The rate of reduced longevity correlates with the severity of AI shown in the affected subjects [23]. The 5-year survival rate in subjects not affected by AI and physiologically structured enamel is about 80% whereas the survival rate in subjects affected by AI is only about 50% [23]. Thus, the rate of necessary replacement of insufficient restorations is about 2.5 times higher than in not affected subjects [23]. Especially recurrent caries (27%) compared to unaffected subjects was the main reason for failure of restorations in subjects affected with AI [23].

In the present case restorations of molars and premolars failed more often than other restorations. Due to the higher occlusal loads of molars and premolars and increased stress within the cervical thirds of these teeth [24] especially approximal margins could show failure of bonding combined with a higher risk of secondary caries. Beside the mechanical higher load also the reduced adhesive bond strength due to the reduced amount of enamel [23] could be responsible for failure. Additionally, the longevity could be reduced probably due to the inferior etching pattern resulting in reduced bond strengths [20]. Beside the reduced bond strength to the enamel also the primary retention to dentin with its less reliable bond strength [20] may be responsible for failure [23]. In the present case the anterior incisors and canines did not show an increased failure rate. Especially in the anterior region the restorations stayed sufficient during the entire follow-up period and the most probable explanation for this finding could be a reduced occlusal load compared to restorations located in the posterior regions. All initial treated teeth could be preserved vital from the first treatment in 2010 till the last follow-up in 2019 after 110 months.

## Conclusion

The performed restorative treatment resulted in an efficient and prompt decrease of the mentioned discomfort and improvement of the quality of life. Treatment was performed minimal invasive and thus contributed to the protection of the pulp without further loss of dental hard tissues. Upon possible breakdown of dentin adhesive restorations these could be repaired by the use of composite resin restorations in order to minimize the risk of pulp exposure upon extensive treatments. Due to the combination of minimal invasive treatment approach with the possibility of repair of composite resin restorations pulp vitality was maintained over the entire observation period and the necessity of more invasive treatment options could be prolonged for nearly 10 years.

## Data Availability

Not applicable.

## Abbreviations

AI: Amelogenesis imperfecta
AOB: Anterior open bite
